# Difference in clinical presentation and surgical outcomes in pediatric and adult patients with Chiari malformation type 1: a single center retrospective study

**DOI:** 10.1007/s00701-025-06534-3

**Published:** 2025-04-24

**Authors:** Erik Öhlén, Victor Gabriel El-Hajj, Victor E. Staartjes, Pascal Jabbour, Erik Edström, Adrian Elmi-Terander

**Affiliations:** 1 Department of Clinical Neuroscience, Karolinska Institutet, Stockholm, Sweden; 2 Center for Clinical Research Sörmland, Uppsala University, Eskilstuna, Sweden; 3 Capio Spine Center Stockholm, Löwenströmska Hospital, Upplands-Väsby, Sweden; 4 Department of Neurosurgery, University Hospital Zurich, Zurich, Switzerland; 5 Department of Neurological Surgery, Thomas Jefferson University Hospital, Philadelphia, USA; 6 Department of Medical Sciences, Örebro University, Örebro, Sweden; 7 Department of Surgical Sciences, Uppsala University, Uppsala, Sweden

**Keywords:** Chiari 1 malformation, Syringomyelia, Chicago Chiari Outcome Scale, Posterior fossa decompression

## Abstract

**Introduction:**

Chiari malformation type 1 (CM1) is a common congenital disorder affecting both children and adults. Although pediatric and adult CM1 patients share many characteristics, the differences between the groups are not fully described.

**Method:**

A comparative analysis was made of two previously defined cohorts of adult and pediatric non-syndromic CM1, surgically treated at the study center. Clinical outcomes were assessed using the Chicago Chiari outcome scale (CCOS) and radiological outcomes were measured as change in cerebellar tonsil and syringomyelia status.

**Results:**

A total of 209 patients (73 pediatric, 136 adults) were included, with median ages of 11 and 33 years, respectively. The proportion of female patients (62% vs 78%) was higher in the adult population (p = 0.012). Headache (p = 0.007), neck pain (p = 0.000), vertigo (p = 0.007), and sensory symptoms (p = 0.000) were more common in adults, while scoliosis (p = 0.000) and sleep apnea (p = 0.015) were more common in the pediatric population. Preoperative imaging findings did not differ significantly. After posterior fossa decompression, both groups scored a median CCOS of 15 at early follow-up (3 vs 4 months), though the pediatric population had a more favorable distribution of CCOS scores (p = 0.003). Postoperatively, syringomyelia status did not differ significantly between groups, but cerebellar tonsil status improved more frequently in adults (64% vs 88%, p = 0.000).

**Conclusion:**

This study demonstrates that while headache is the most common presenting symptom in both pediatric and adult CM1 patients, pediatric patients are more likely to present with scoliosis and sleep apnea. In contrast adult patients more frequently experience headache, neck pain, vertigo, and sensory symptoms. There were no differences in other preoperative imaging variables and outcomes were favorable for most patients in both groups.

## Introduction

Chiari Malformation type 1 (CM1) is a congenital disorder of the hindbrain, characterized by a herniation of the cerebellar tonsils into the foramen magnum. The resulting overcrowding in the foramen magnum leads to a disturbed flow of cerebrospinal fluid (CSF) and compression of nearby neural structures. This, in turn may lead to the development of hydrocephalus and syringomyelia [17, 18, 27, 47, 55]. While embryologic underdevelopment of the occiput has been suggested to underlie the pathology, the definitive pathophysiology of CM1 remains to be elucidated [8, 42, 47, 52]. The radiological definition of CM1, a descent of the cerebellar tonsils > 5 mm below foramen magnum, is met by 0.9% of the general population [11, 53]. However, incidental findings are not uncommon and only 1 in 1000 individuals present with an overt clinical condition [35].

The condition presents in both children and adults, with a median peak age of 8 years among children and 41 in adults [6]. CM1 may involve a wide range of symptoms, including exertion-related headache, neck pain, sensory and motor deficits, dysphagia, sleep apnea and cognitive impairment [4, 35]. Posterior fossa decompression (PFD), with or without duraplasty, is the treatment of choice for symptomatic patients [33, 39, 46]. The postoperative outcome is usually favorable, and around 75% of patients experience improvement in symptoms after surgery [3, 5, 6, 33].

Although pediatric and adult CM1 patients share many characteristics, some differences between the groups have been described but are yet to be completely understood.

Pediatric CM1 patients more commonly present as either asymptomatic or with scoliosis, impaired oropharyngeal function and central sleep apnea. However, as with adult patients, exertion-related headache is still the most common symptom[2, 12, 23]. Adult patients develop sensory- and motor deficits more frequently than pediatric patients [36, 40]. In some studies, the presence of syringomyelia is more common in adults [2, 6, 19] while in others it is more common in pediatric patients [40]. The same conflicting reports also apply for postoperative outcomes [6, 19, 36, 40]. These contradictory results prompt further investigation. The aim of this retrospective study of pediatric and adult CM1 patients was to further elucidate differences in characteristics and outcome between the two populations.

## Methods

### Study design and patient selection

This was a single-center retrospective population-based cohort study of pediatric and adult patients who were surgically treated for CM1 malformation. This study utilizes the same adult and pediatric CM1 cohorts that have previously been described in studies from our institution [15, 33].

The study hospital is a publicly funded and owned tertiary care center that serves a region of about 2.3 million inhabitants with neurosurgical care. Patients were identified in the surgical management software Orbit (Evry Healthcare Systems, Solna, Sweden). Medical records and the imaging data were retrospectively reviewed using the health record software TakeCare (CompuGroup Medical Sweden AB, Farsta, Sweden).

The above-mentioned studies included all pediatric (< 18 years) and adult (≥ 18 years) patients who received a PFD due to non-syndromic CM1 between 2005 and 2020 and 2005 and 2017, respectively.

### Variables

Baseline patient characteristics included sex, age at time of surgery, Body Mass Index (BMI), and presenting symptoms and findings. All patients had a preoperative magnetic resonance imaging (MRI) of the brain and spine, and imaging data included the presence of tonsillar herniation, the grade of cerebellar tonsillar descent (CTD), the presence of syringomyelia and hydrocephalus. Tonsillar herniation was defined as a descent of the cerebellar tonsils > 5 mm below foramen magnum. CTD was graded as previously suggested; grade 1: tonsils descend through the foramen magnum without reaching the C1 arch – grade 2: tonsils descend to the level of the C1 arch—grade 3: tonsils descend below the C1 arch [58]. The Chiari Severity Index (CSI) was calculated for all patients based on the imaging findings [22]. Evaluations were made of the surgical technique, surgical complications (according to the Landriel-Ibanez grading scheme [34]), and the need for reoperation.

### Outcome

The outcomes were evaluated clinically and radiologically at early (3–4 months) and, when applicable, late follow-up (latest available data). Pediatric patients are routinely followed until 18 years of age, while adult patients have a postoperative follow-up at three months. Later follow-ups are performed if and when clinically indicated. For the clinical outcome, the Chicago Chiari Outcome Scale (CCOS) [3], was used for evaluation. CCOS takes four postoperative outcome measures into consideration: pain symptoms, non-pain symptoms, functionality and complications. Pain symptoms include headache, neck pain and dysesthesia in the extremities. Non-pain symptoms include vertigo, sensory loss, motor deficits, dysphagia and other neurological signs. Functionality is defined as the patient´s ability to attend their daily responsibilities, such as work or school. Complications include all surgical complications. Possible scores were 4–16, and scores ≤ 8 were considered as unfavorable outcome, scores between 9–12 were considered as unchanged outcome and scores ≥ 13 were improved outcome. Radiological outcome was measured as change in the cerebellar tonsil herniation, where an ascent of the tonsils closer to the foramen magnum was considered as an improvement and a measurement of ≤ 5 mm below foramen magnum was considered as complete improvement. Change in syringomyelia size was also evaluated as a radiological outcome, with a reduction in the anterior-poster-diameter considered an improvement, and full resolution of the syringomyelia regarded as complete improvement.

### Statistics

Medians and interquartile ranges (IQRs) were used to describe continuous data, while categorical data were presented using numbers and proportions. Chi-squared and, when appropriate, the Fisher’s Exact tests were used to compare categorical data between groups, while the Mann–Whitney U test was used to compare continuous data across binary groups. Statistical analyses were performed using IBM SPSS Statistics, version 26, and p-values of < 0.05 were considered statistically significant. Figures and tables were created using statistical software R, version 4.4.2, and Microsoft Excel, version 2412.

## Results

### Baseline characteristics

A total of 209 patients were included in the study. At the time of surgery, 73 were pediatric (< 18 years), 136 were adults (≥ 18 years) and the median ages were 11 years (IQR 6–14, range 11 months to 17 years) and 33 years (IQR 23–46, range 18 to 81 years), respectively. Most patients were female (72%). The proportion of females was significantly higher (p = 0.012) in the adult (78%) compared to the pediatric (62%) group. The median BMI was 17 (IQR 16–21) for children and 25 (IQR 22–28) for adults (Table 1).

**Table 1:** Patient Characteristics

	Total, N = 209	Pediatric, N = 73	Adult, N = 136	p-value
Sex (female)	151 (72%)	45 (62%)	106 (78%)	**0.012**
Age at operation	22 (14–36)	11 (6–14)	33 (23–45)	**-**
BMI	23 (19–27)	17 (16–21)	25 (22–28)	**-**
**Presenting symptoms and findings**				
Headache	155 (74%)	46 (63%)	109 (80%)	**0.007**
Neck pain	100 (48%)	15 (21%)	85 (63%)	**0.000**
Vertigo or impaired balance	77 (37%)	18 (25%)	59 (43%)	**0,007**
Numbness/tingling	71 (34%)	12 (16%)	59 (43%)	**0.000**
Visual	30 (14%)	12 (16%)	18 (13%)	0.529
Scoliosis	25 (12%)	25 (34%)	0 (0%)	**0.000**
Motor deficit	23 (11%)	9 (12%)	14 (10%)	0.654
Dysphagia	11 (5%)	3 (4%)	8 (5%)	0.584
Auditory	9 (4%)	4 (6%)	5 (4%)	0.540
Sleep apnea	8 (4%)	6 (8%)	2 (2%)	**0.015**
Bowel/bladder	2 (1%)	1 (1%)	1 (1%)	0.653
**Imaging findings**				
Tonsillar herniation	206 (99%)	72 (99%)	134 (99%)	0.953
Cerebellar Tonsil descent (CTD) (1 missing)				0,114
0 (tonsils < 5 mm below FM)	3 (1%)	2 (3%)	1 (1%)	
1 (> 5 mm below FM)	26 (13%)	9 (12%)	17 (13%)	
2 (at C1 arch)	120 (58%)	34 (47%)	86 (64%)	
3 (below C1 arch)	59 (28%)	28 (38%)	31 (23%)	
Syringomyelia	85 (41%)	33 (45%)	52 (38%)	0.328
Hydrocephalus	8 (4%)	5 (7%)	3 (2%)	0.095
**Presenting clinical score**				
Chiari severity index (CSI)				0.233
1	102 (49%)	29 (40%)	73 (54%)	
2	55 (26%)	27 (37%)	28 (21%)	
3	52 (25%)	17 (23%)	35 (26%)	

Values are presented either as the number of patients (%) or medians (IQR). Abbreviations: BMI = body mass index, FM = foramen magnum.

### Presenting symptoms and findings

The main symptom preceding surgery was headache for both groups (63 and 80%), followed by neck pain for adults (63%) and scoliosis for children (34%). Headache, neck pain, vertigo, and numbness/tingling were significantly more common (p < 0.05) in the adult group and scoliosis and sleep apnea were more common in the pediatric group. The frequencies of visual and auditory symptoms, as well as motor deficits and dysphagia, were not significantly different between the two groups. Among the rarer symptoms were orthostatic syncope (7 patients), bladder and bowel dysfunction (2 patients), epileptic seizures (2 patients), abducens paresis (1 patient), ataxia (1 patient) and drop attacks (1 patient). Of the patients experiencing these symptoms, all except one (a child with bladder and bowel dysfunction) were adults.

On preoperative imaging, most of the patients showed tonsillar herniation of > 5 mm (99% of children and 99% of adults) and a CTD grade of 2 (47 and 64%, respectively). Syringomyelia was present in 33 (45%) of the pediatric patients and in 52 (38%) of the adult patients. Hydrocephalus was found in 5 (7%) of the pediatric patients and in 3 (2%) of the adult patients. Only one of them (a child) received hydrocephalus surgery (endoscopic third ventriculostomy (ETV)) prior to treatment of the Chiari malformation. The reason why the other patients with hydrocephalus did not receive hydrocephalus surgery prior to PFD could not be deduced from the patient charts. Notably, the patient treated with ETV was treated in 2017 and the other patients were treated between 2005 and 2016, possibly suggesting changes at the study center in the diagnostic workup or surgical decision making. None of the findings in the preoperative imaging except scoliosis were significantly different between the two groups (Table 1).

**Fig. 1:**
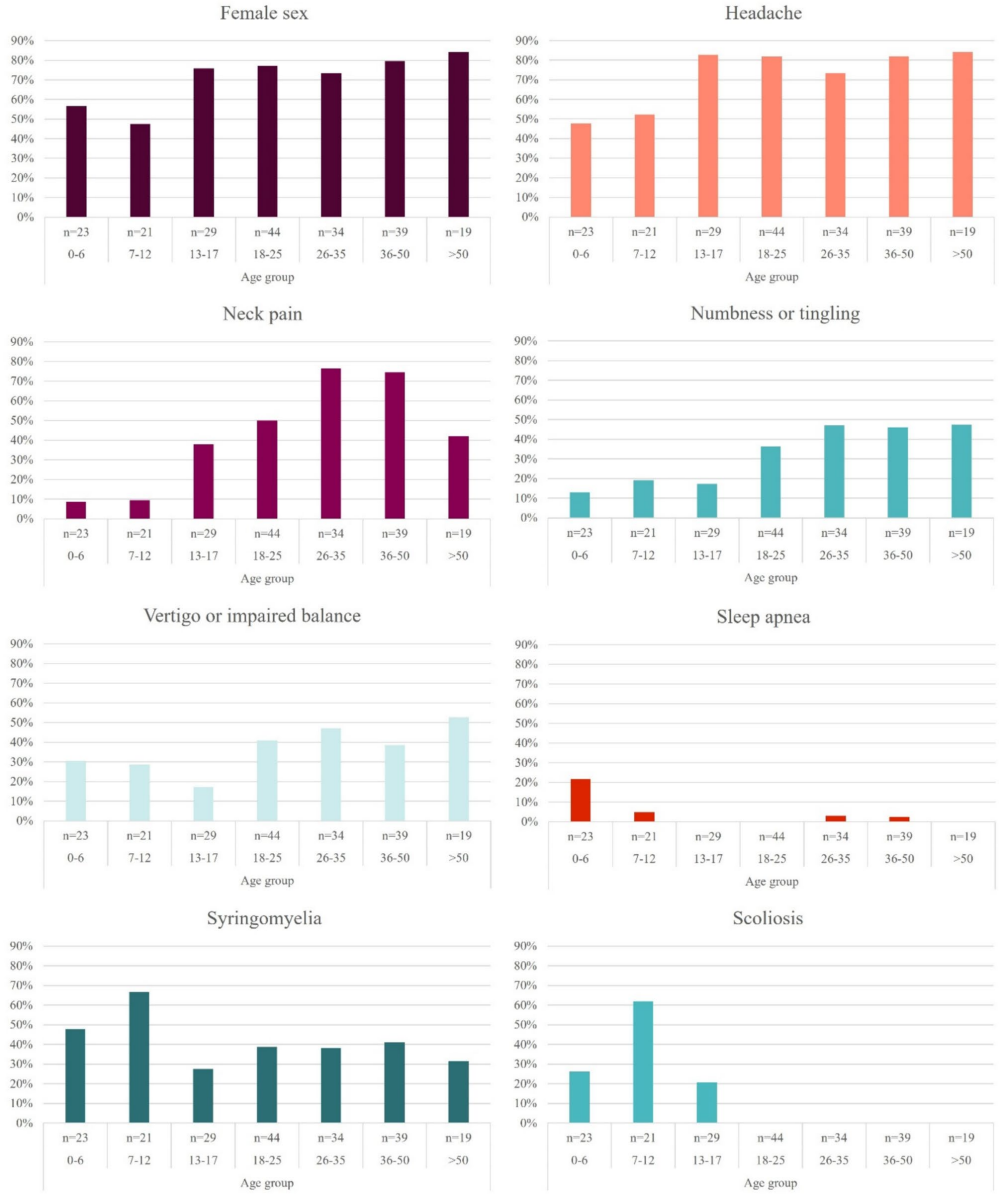
Relative distribution of patient characteristics across age groups

To further elucidate patterns of patient characteristics across different ages, patients were divided into even age groups and frequencies were calculated for each patient characteristic. Common and statistically significant characteristics are presented in Fig. 1.

### Surgical data and complications

All patients underwent PFD and the absolute majority of patients received a duraplasty (98%). The hospital stay was slightly longer for the pediatric population (p < 0.024), with a median of 6 days (IQR 5–8) compared to 5 (IQR 4–7).

Complications were reported for 20% of all patients and the complication rate was not significantly different between the two groups. There was also a similar distribution in Ibanez gradings between the groups, where grade 1b (requiring pharmacological treatment) was the most common (48% of all complications). In total, meningitis was the most common complication (6%) (of which all but one was considered as chemical meningitis) which was also true for the pediatric population, but CSF leak was slightly more common in the adult population (7%). However, new postoperative or worsened hydrocephalus was the only complication which was significantly different between the populations (p = 0.041), and it occurred in three of the pediatric patients. Of these, one did not have prior hydrocephalus, and the postoperative hydrocephalus observed was transient. The other two received ventriculoperitoneal (VP) shunts for their worsening hydrocephalus (Table 2).

**Table 2:** Surgical data and complications

	Total, N = 209	Pediatric, N = 73	Adult, N = 136	p-value
**Surgical technique**				0.679
Decompression only	3 (1%)	1 (1%)	2 (2%)	
Decompression and opening of dura	1 (1%)	0 (0%)	1 (1%)	
Decompression and dura graft	205 (98%)	72 (99%)	133 (98%)	
**Complications**				
30-day complications	42 (20%)	15 (21%)	27 (20%)	0.905
Ibanez grade				0.923
1a (spontaneously resolving)	8 (4%)	3 (4%)	5 (4%)	
1b (requiring pharmacological treatment)	20 (10%)	7 (10%)	13 (10%)	
2a (requiring invasive treatment without general anesthesia)	7 (3%)	3 (4%)	4 (3%)	
2b (requiring invasive treatment with general anesthesia)	6 (3%)	2 (3%)	4 (3%)	
3a (requiring management in intensive care unit)	1 (1%)	0 (0%)	1 (1%)	
**Complications specified**				
Meningitis	13 (6%)	6 (8%)	7 (5%)	0.383
CSF leak	12 (6%)	3 (4%)	9 (7%)	0.548
Hematoma/hygroma	8 (4%)	3 (4%)	5 (4%)	1.000
Wound infection	6 (3%)	1 (1%)	5 (4%)	0.667
New or worsened Hydrocephalus	3 (1%)	3 (4%)	0 (0%)	**0.041**
Other complication	3 (1%)	2 (3%)	1 (1%)	0.280
Urinary tract infection	2 (1%)	1 (1%)	1 (1%)	1.000
Length of hospital stay, days	6 (5–7)	6 (5–8)	5 (4–7)	**0.024**
Reoperation	19 (9%)	9 (12%)	10 (7%)	0.233

Values are presented either as the number of patients (%) or medians (IQR). Abbreviations: CSF = cerebrospinal fluid.

### Follow-up and outcomes

The initial clinical follow-up was carried out at a median of 3 months (IQR 2–4) for the pediatric population and 4 months (IQR 3–5) for the adult population. Three adult patients had no clinical follow-up; one moved to another city and two were only followed with MRI. As per routine in the study clinic, most pediatric patients had long term follow-ups (89%, median 100 months, IQR 73–124) whereas only a minority of adult patients received longer follow ups (22%, median 19 months, IQR 10–41) than the standard initial follow up at 3 months.

92% of all patients had a postoperative MRI performed during follow-up, and the first MRI was performed at a median of 4 months (IQR 2–8, 17 missing) after surgery for the pediatric population and at 3 months (IQR 3–4, 10 missing) for the adult population.

At early follow-up, both the pediatric and adult population reached a median CCOS score of 15 (IQR 15–16 and 15–16, respectively). An absolute majority of patients scored ≥ 13 on CCOS (93%), which was considered as improved and very good outcomes. Among patients who scored 9–12 on CCOS (unchanged outcomes), there was a slight predominance of adults (8 vs 5%) and the distribution of CCOS scores between the two groups was significantly different (p = 0.003). No patients scored below 10 at initial follow-up.

Regarding the radiological outcome, early postoperative MRI showed a completely or partially improved syringomyelia status in 73% of all patients with preoperative syringomyelia.

As previously reported, four patients in the pediatric population had worsened syringomyelia status, with two of them developing a new syringomyelia postoperatively. An additional patient in the pediatric population developed a new syringomyelia at long-term follow-up, without an apparent or identifiable cause and which did not require additional surgery [15].

There was a significant difference in early postoperative tonsil status between the groups, where the adult population showed a higher proportion of complete or partial reduction of tonsillar herniation (88 versus 64%, p = 0,000). Two patients in each group showed a worsening tonsillar herniation. The two pediatric patients and one of the adult patients underwent revision surgery.

Including the aforementioned patients, a total of 19 patients (9%) required revision surgery, with no significant differences observed between the groups. The indications for revision surgery were, with the exception of worsened hydrocephalus (described above under complications), similar and included recurrence of symptoms, progression of tonsillar herniation and worsened or unchanged syringomyelia status.

A late postoperative MRI was performed in 47 of the pediatric patients and 25 of the adult patients, at a median of 59 (IQR 36–90) and 21 months (IQR 15–47), respectively.

The long term clinical follow up of the pediatric patients, has previously been reported [15]. In the adult population, however, long-term follow-ups were performed based on clinical indications such as worsening of symptoms or complications. Therefore, a systematic comparison to the pediatric group is not possible. The long-term outcomes for the adult population are described below.

### Long term follow-up of adult patients

Long term follow-ups were performed in 30 of the adult patients (22%). The main reasons for adult patients requiring longer follow-ups were remaining symptoms, complications and unsatisfactory results on initial postoperative MRI. Due to the small proportion of adult patients receiving longer follow-ups, no further statistical comparisons were made with the pediatric population to avoid selection bias (Table 3).

**Table 3:** Clinical and radiological outcomes at early follow-up

	Total	Pediatric	Adult	p-value
**Clinical follow-up**	n = 206	n = 73	n = 133	
Months after surgery	4 (3–5)	3 (2–4)	4 (3–5)	
CCOS	15 (14–16)	15 (15–16)	15 (14–16)	**0.030**
**Radiological follow-up**	n = 179	n = 56	n = 123	
Months after surgery	3 (3–5)	4 (2–8)	3 (3–4)	
**Syringomyelia status**	n = 77	n = 32	n = 45	0.150
Improved	58 (75%)	20 (59%)	38 (84%)	
Complete Improvement	6 (8%)	2 (6%)	4 (9%)	
Partial Improvement	52 (68%)	18 (53%)	34 (76%)	
Unchanged	17 (22%)	10 (29%)	7 (16%)	
Worse	2 (3%)	2 (6%)	0 (0%)	
New onset postoperative syringomyelia	2 (2%)*	2 (4%)*	0 (0%)	
**Cerebellar tonsil status**	n = 180	n = 56	n = 124	** < 0.001**
Improved	145 (81%)	36 (64%)	109 (88%)	
Complete improvement	97 (54%)	30 (54%)	67 (54%)	
Partial improvement	48 (27%)	6 (11%)	42 (34%)	
Unchanged	31 (17%)	18 (32%)	13 (10%)	
Worse	4 (2%)	2 (4%)	2 (2%)	

Values are presented either as the number of patients (%) or medians (IQR). n = numbers of patients with data available. *out of patients with no preoperative syringomyelia.

## Discussion

In this retrospective, single center, population-based cohort study we compared characteristics and outcomes between surgically treated pediatric and adult CM1 patients. An absolute majority of patients in both groups showed a favorable outcome, both clinical and radiological. However, our results showed differences between the groups which require further discussion.

In our study population, there was a predominance of female patients both in the pediatric and adult groups. However, the adult population showed a greater skew toward female predominance (78 versus 62%). This is in line with previous studies [7, 19, 40], although the reason for this is poorly understood. Mortazavi et al. [40] suggest that CM1 may coexist with hypermobility in older female patients, and since hypermobility is more common among females, this would explain the female predominance. CM1 has indeed been reported as a comorbid condition of connective tissue disorders, and the craniocervical hypermobility seen in these patients has even been suggested as a possible pathophysiological mechanism for CM1. However, a known connective tissue disorder is present in only 1% of CM1 patients [10], making it unlikely to be the sole reason for female predominance in CM1 patients. In our material (Fig. 1), the skew towards female predominance starts already in adolescence (> 12 years). Whether this stands for increasing hypermobility as the patient becomes older or other comorbidities is unclear and needs to be further studied.

Regarding differences in the presentation of symptoms, headache, neck pain, vertigo, and numbness or tingling were significantly more common in the adult group, and scoliosis and sleep apnea were more common among pediatric patients. In the literature, headache is found in about 80% of adult CM1 patients [40, 51] and in approximately 50% of pediatric patients [2, 9, 40], which is in line with our data (80 vs 63%). The explanation for this difference is not entirely understood and it has been suggested that symptoms develop gradually with increasing age in the pediatric population [2]. Another explanation for headache being more common among adults could be a comorbidity with other headache conditions, such as migraine or tension headaches [50]. This would be in line with a review by Arnautovic et al. [6], where they could show a significant difference in the improvement or resolution of headache in favor of the pediatric population, indicating that another explanation for headache in the adult population was present. Importantly, in the youngest children clinical diagnosis must rely on objective parameters since they cannot reliably provide information on symptoms such as headache, vertigo, numbness or tingling. This becomes evident when studying symptom presentation across age groups (Fig. 1), where the prevalence of headache and neck pain among adolescents (ages 13–17) aligns closely with that observed in the adult patients.

In children, conditions affecting the spinal cord may promote the development of scoliosis [44]. While there is a well-established association between CM1, syringomyelia and scoliosis, there is less evidence regarding a proposed genetic connection [20, 31, 48]. Animal studies have demonstrated that several signaling pathways linked to the development of congenital and idiopathic scoliosis, also play a role in embryonic neurulation and the formation of CSF circulation [24, 26]. In CM1 patients younger than 10 years, surgical treatment of the Chiari malformation has been shown to reduce the spinal deformity, omitting the need for further orthopedic intervention, [43]. In this study, the highest prevalences of syringomyelia and scoliosis were both found in patients 7 to 12 years old (67% and 62%, respectively) (Fig. 1). A further exploration of the relationship between CM1 and scoliosis would be of interest but lies outside the scope of this study. Other than the presence of scoliosis, there were no significant differences in preoperative imaging findings between the groups in our study population. This is interesting from a pathophysiological point of view, especially regarding the presence of syringomyelia. In their large systematic review from 2014, Arnautovic et al. [6] analyzed 145 published series and found incidences ranging from 12–100% with a higher presence of syringomyelia in adult CM1 patients (69 vs 40%). To explain this, they argue that symptomatic CM1 and syringomyelia take time to develop. The frequency of spontaneous resolution of syringomyelia occurring secondary to CM1 is unknown but if most cases remain until surgically treated the frequency in an untreated population would be expected to increase with age.

Sleep apnea was more common among the pediatric patients (8 vs 2%), especially in the youngest age group (ages 0–6) (22%). Although most clinicians would agree CM1 may cause central sleep apnea, the reported prevalences of sleep apnea in CM1 vary considerably. In contradiction to our study, Mortazzavi et al. [40] could show a higher prevalence of sleep apnea in adult patients (15 vs 7%), and in a study on adult CM1 and 2 patients the prevalence was as high as 59% [29]. Kirjavainen et al. [32] could show a high prevalence (45%) of central sleep control disorder in pediatric CM1 patients, although severe sleep apnea was deemed rare and only 18 out of 104 patients required decompressive surgery. In agreement with our study, central sleep apnea has also been shown to be more common among younger children (< 6 years) [23, 41]. The pathophysiological mechanism behind sleep apnea in CM1 is stipulated to be a compression of the brainstem, resulting in an impaired central ventilatory drive [32]. Why this would be more common among younger children is unclear, but evidently sleep apnea needs to be taken into consideration when evaluating pediatric Chiari patients in the clinic.

In this study, almost all patients were operated with PFD and duraplasty (98%). Complications occurred in 20% of the patients, and the most reported complications were meningitis and CSF leak (both 6%). This is in line with previous studies. Greenberg et al. reported a complication rate of 19% in adult patients receiving PFD with duraplasty and Durham et al. showed a 19% rate of CSF-related complications for pediatric patients receiving PFD with duraplasty in a meta-analysis [13, 21]. The only complication that differed significantly between the populations was new-onset or worsened hydrocephalus which occurred in 3 (4%) of the pediatric patients and none of the adult patients. In the literature, a preoperative hydrocephalus is present in 7–10% of patients and is an independent predictor of postoperative morbidity [14, 21, 45]. Hydrocephalus occurs as a complication in 1–9% of surgeries and is reportedly slightly more common in pediatric than adult patients [6, 25, 45]. It should be noted that nowadays, the standard procedure is to manage the hydrocephalus in a Chiari patient before performing a PFD [38].

CCOS is a validated outcome score for both pediatric and adult CM1 patients [3, 5]. Considering the postoperative outcome of symptoms (pain and non-pain), functionality, and complications, it gives a comprehensive representation of the patient’s clinical outcome. The functionality component of CCOS has been validated in adult populations [5], but its relevance in assessing outcomes for pediatric CM1 patients has been questioned [1, 57]. As a result, a modified CCOS excluding this component has been proposed for pediatric patients. However, this modified version has demonstrated limited variability. Given that functionality is a key concern in postoperative outcomes for adults, we opted to use the same CCOS version for both populations, consistent with previous studies comparing pediatric and adult populations [19].

In this study, 95% of pediatric and 93% of adult patients scored ≥ 13 on CCOS at initial follow-up which was considered good outcomes. However, the distribution of CCOS scores was different between the groups, with lower scores for adult patients (p = 0.003). No comparisons of outcome at long-term follow-up could be made between the groups due to the lack of data in the adult population (as described in the results section). Gilmer et al. could in a study on both pediatric and adult CM1 patients show a general decrease in CCOS over time for the adult patients (especially patients > 40 years), and a stable CCOS score over time for the pediatric patients [19]. The reason for this is unclear and studies with long-term follow-ups are needed.

Radiological outcome, expressed as an improvement of syringomyelia and cerebellar tonsil status, was favorable for both groups. On the initial postoperative MRI, a majority of the adult and the pediatric patients experienced partial or complete improvement in the syringomyelia status, which is in line with the literature [6, 40]. The adult population showed a greater improvement in cerebellar tonsil status on initial MRI than the pediatric population (88 vs 64%, p < 0.001). In a long-term study on 42 pediatric CM1 patients, Massimi et al. could show an improvement in tonsil status in 50% of their patients after surgery [37]. Their population had, however, a lower rate of improvement on CCOS (70%). In a large long-term study on 297 adult patients, a favorable CCOS outcome in 63% did not correlate to an improvement in tonsil status which was seen in only 18% [54]. In our earlier study of the pediatric population, no correlations between early tonsillar or syringomyelia status and outcome were seen [15]. Other studies on the correlation of syringomyelia status and clinical outcome in both pediatric and adult patients have shown similar results [16, 49]. Complete postoperative resolution of tonsillar and syringomyelia status may take months or years and seems to vary greatly between individuals [28, 56]. Hu et al. could show older age as a predictor of worse clinical outcome and a lower rate syrinx resolution, arguing that longer standing central canal enlargement and aging decreases the likelihood of syrinx resolution [30]. This is supported by Gilmer et al., who argue that aging leads to tissue inflexibility, thereby decreasing the chance of the central canal returning to its original state [19]. Our results contradict these arguments, since the adult population showed a great degree of postoperative improvement in syringomyelia status. While CM1 may be defined using radiological parameters, it is important to recognize that the symptoms relate to obstruction of CSF-flow and compression of neural structures. Once a PFD is performed to resolve these problems the relative position of the tonsils have little or no clinical consequence. Similarly, the resolution of a syringomyelia after a PFD must be dependent on other factors than direct compression.

## Strengths and limitations

The main strength of this study is its population-based design. By studying patients from a single neurosurgical center serving a region of 2.3 million inhabitants, it provides a representative overview of all CM1 patients (both pediatric and adult) in need of surgical intervention, facilitating a robust comparison between these groups. However, this may also be viewed as a limitation, as the findings reflect the clinical decisions of a small group of surgeons, which can introduce bias. Another limitation is the lack of long-term follow-up data for the adult patients, which limits the possibility to compare long-term outcomes with the pediatric population. The study is retrospective, which may introduce reviewer bias during chart reviews and is dependent on the quality of the available clinical data.

## Conclusions

This study demonstrates that while headache is the most common presenting symptom in both pediatric and adult CM1 patients, pediatric patients are more likely to present with scoliosis and sleep apnea. In contrast adult patients more frequently experience headache, neck pain, vertigo, and sensory symptoms. There were no differences in other preoperative imaging variables and the clinical and radiological outcomes were favorable for most patients in both groups.

## Data Availability

The data that support the findings of this study are not openly available due to reasons of sensitivity and are available from the corresponding author upon reasonable request. Data are located in controlled access data storage at Karolinska Institutet.

## Abbreviations

CM1: Chiari malformation type 1
CCOS: Chicago Chiari outcome scale
CSF: Cerebrospinal fluid
PFD: Posterior fossa decompression
MRI: Magnetic resonance imaging
BMI: Body mass index
CTD: Cerebellar tonsillar descent
CSI: Chiari severity index
IQR: Interquartile range
ETV: Endoscopic third ventriculostomy
FM: Foramen magnum
VP: Ventriculoperitoneal
